# High voltage generation from wastewater by microbial fuel cells equipped with a newly designed low voltage booster multiplier (LVBM)

**DOI:** 10.1038/s41598-020-75916-7

**Published:** 2020-11-04

**Authors:** N’Dah Joel Koffi, Satoshi Okabe

**Affiliations:** Division of Environmental Engineering, Faculty of Engineering, Hokkaido University, North-13, West-8, Kita-ku, Sapporo, Hokkaido 060-8628 Japan

**Keywords:** Renewable energy, Civil engineering

## Abstract

Although microbial fuel cells (MFCs) can produce renewable energy from wastewater, the generated power is practically unusable. To extract usable power from an MFC fed with wastewater, we newly developed a low voltage booster multiplier (LVBM), which is composed of a self-oscillating LVB and multistage voltage multiplier circuits (VMCs). The low output MFC voltage (ca. 0.4 V) was successfully boosted up to 99 ± 2 V, which was the highest voltage that has been ever reported, without voltage reversal by connecting an LVB with 20-stage VMCs. Moreover, the boosted voltage (81 ± 1 V) was stably maintained for > 40 h even after disconnecting the LVBM from the MFC. The energy harvesting efficiency of LVBM was > 80% when an LVB with 4-stage VMCs was charged to 9.3 V. These results clearly suggest that the proposed LVBM system is an efficient and self-starting energy harvester and storage for low-power generating MFCs.

## Introduction

Microbial fuel cells (MFCs) are bio-electrochemical devices that can directly convert chemical energy in biodegradable organic matter to electrical energy by exoelectrogenic bacteria as catalyst. In MFCs, exoelectrogenic bacteria extract electrons from the energy source (simply oxidation) and transfer them to the anode through diverse extracellular electron transfer (EET) mechanisms (so-called anodic respiration). The resulting electrons are then transported to the cathode and used for the reduction reaction of oxidized compounds (i.e., oxygen in the case of air–cathode MFC), which generates electrical power^1^. If wastewater is used as an energy source, simultaneous wastewater treatment and renewable energy production can be achieved. Therefore, it is expected that MFC technology is a promising energy-positive wastewater treatment process. However, the power generated from individual MFCs is practically unusable (unable to directly drive even low-power electronic devices such as LEDs and wireless sensor radios) due to its high internal resistance and low voltage output, which is a current major issue of MFC technology. The required operating voltage of such devices ranges from 2 to 5 V at least, and the power consumption could be up to the order of 1 W^2^. The maximum theoretical voltage across anode and cathode (*E*^*0*^_cathode_ − *E*^*0*^_bioanode_) of a single air–cathode MFC is 1.14 V (assuming *E*^*0*^_bioanode_ = *E*^*0*^_NADH_ = − 0.32 V and *E*^*0*^_cathode_ = *E*^*0*^_oxygen_ =  + 0.82 V vs. SHE at neutral pH, respectively)^3^. However, a typical observed open-circuit voltage (OCV) of an air–cathode MFC is in a range of 0.2–0.5 V due to the losses of electrode potential, such as activation polarization, concentration polarization, and Ohmic losses^4^, which are also dependent on substrate, microorganisms, electrode overpotentials, internal resistance and applied external resistance^5,6^. The power density that an MFC can typically generate is from 1 to 2000 mW m^−2^^4^. Therefore, the MFC output voltage and power must be increased for practical uses.

So far, several MFCs were simply connected in series or in parallel to overcome the low voltage or power issue. However, although a serially stacked MFCs unit could provide a higher voltage, it has often been proven to be difficult and ineffective due to voltage reversal (reverse polarity owing to fuel shortage) of individual MFC units, leading to a significant overall voltage decay^2,5–7^. Recently, significant efforts have been made to control and suppress the voltage reversal occurrence by connecting individual MFC units with a maximum power point tracking (MPPT) system to charge a stacked polarized capacitor^8,9^. However, this technical approach could increase stacked voltages only within the range of 2 to 3 V^8,9^.

Alternatively, power management systems (PMSs) have been proposed to boost the low MFC output voltage to a certain required voltage and store enough energy to power the electronic loads. The PMSs basically utilize a combination of a direct current to direct current (DC/DC) converter to boost low MFC voltage to usable levels and a supercapacitor to store electrical energy temporarily. To date, various types of commercially available or individually developed PMSs were proposed to interface MFCs with electronic loads, and their performances were evaluated^2,6,7,10–16^ (Table 1). We have also successfully achieved a boosted voltage of 5.2 V from wastewater by a single-chamber air–cathode MFC equipped with a low voltage booster (LVB)^16^. Two units of an MFC equipped with an individual commercially available PMS were connected in series, which could generate only a final stacked voltage of 6.6 V^6^. In these studies, low MFC output voltages were increased to 2–12 V, which could drive only low voltage electronic devices but still not enough for real-world applications. Most of the existing PMSs for MFC applications are manufactured to only boost the low voltage, not further amplify or multiply the boosted voltage. Accordingly, to further increase the output voltage, a secondary voltage multiplier or amplifier of the primary boosted voltage is required.

**Table 1:** Comparison of previously reported MFC equipped with commercially available or individually developed power management systems (PMSs). UPEM: Ultra-low power energy for MFC; IC: Integrated circuit; LVBM-[n]: Low voltage booster multiplier with the cascade multiplier stage number [n]. N/A: Not available.

PMS	MFC reactor Type	MFC working voltage	MFC-stacked configuration	Output boosted voltage	Efficiency (%)	Refs
DC-DC voltage booster circuit	Two-chamber MFCs (1.2 mL)	0.2–0.4 V	3 MFCs unit in parallel	> 3 V	N/A	^20^
Blocking oscillator booster circuit	Two-chamber MFCs (120 mL)	0.837 V	3 MFCs unit in series	~ 3 V	N/A	^10^
DC–DC voltage Boost converter	Two-chamber MFCs (50 mL)	< 0.2 V	Single or 4 parallel MFCs unit	3.3 V	N/A	^6^
Capacitor-based DC-DC converter	Air–cathode MFC (27 mL)	~ 0.5 V	4 parallel MFCs unit	2.5 V	N/A	^7^
Capacitor-based DC-DC converter	Sediment Microbial Fuel Cells (550 mL anolyte, 445 mL catholyte)	0.4 V	Single MFC unit	3.3 V	N/A	^11^
Synchronous Flyback converter	Two-chamber MFCs (150 mL)	0.310 V	Single MFC unit	2.13 V	50.3	^12^
Diode-based Boost-converter	Two-chamber MFCs (150 mL)	0.311 V	Single MFC unit	1.83 V	22	^12^
DC–DC voltage Boost converter + supercapacitor	Single-chamber air–cathode MFC (316 mL)	0.3 V	Single MFC unit	3.3 V	5.33	^2^
Flyback converters	Benthic MFC	0.35–0.5 V	Large scale benthic MFC	12 V	77	^13^
Flyback converters	Air–cathode MFC (450 mL)	0.44 V	Single MFC unit	5 V	26	^14^
Transformer-based booster + supercapacitor	Single-chamber air–cathode MFC (316 mL)	0.18 V	Single MFC unit	3.3 V	N/A	^22^
LVB	Single-chamber air–cathode MFC (300 mL)	0.2–0.5 V	Single MFC unit	5.2 V	N/A	^16^
UPEM boost converter with one storage capacitor and switch IC	Sediment MFC (15 L)	0.2 V	Single MFC unit	3.3 V	28–36.5	^15^
LVBM-[4]	Single-chamber air–cathode MFC (300 mL)	0.2–0.5 V	Single MFC unit	17 V	82–83	This study
LVBM-[8]	Single-chamber air–cathode MFC (300 mL)	0.2–0.5 V	Single MFC unit	35 V	N/A	This study
LVBM-[20]	Single-chamber air–cathode MFC (300 mL)	0.2–0.5 V	Single MFC unit	99 V	N/A	This study

In this study, we therefore propose a new two-step “boost-and-multiply” system, in which the low MFC output voltage is firstly boosted into an alternating current (AC) voltage by a transistor-based self-oscillating low voltage booster circuit^16^. After, the boosted AC voltage is further multiplied and turned back into a direct current (DC) voltage by a multistage Single-Phase Cockroft-Walton voltage multiplier circuit (VMC)^17,18^. We have manufactured a low voltage booster multiplier (LVBM) and tested its performance using a single-chamber air–cathode MFC treating domestic wastewater as a power source. A low voltage booster with a 20-stage alternating current to direct current (AC/DC) voltage multiplier circuit was able to amplify the MFC voltage (ca. 0.4 V) up to 99 ± 2 V and retain for a few days without voltage reversal, which was the highest boosted voltage that has ever been reported. The feasibility of LVBM application in MFC technology is discussed.

## Materials and methods

### Air–cathode MFC setup and operation

A single-chamber air–cathode MFC was made from an acrylic block (20 × 18 × 3 cm^3^) and composed of the serpentine flow field with a working volume of 0.3 L. The MFC system consists of 4 anode graphite fiber brushes [2.2 cm (diameter) × 12 cm (length), Mill-Rose, Mentor, OH] installed in 4 flow channels, which are sandwiched with 2 separator electrode assembly (SEA) cells as previously described^16^. MFC had two-sided polyvinylidene fluoride (PVDF)-based activated carbon air-cathodes. The PVDF-based activated carbon air-cathodes (10 cm × 10 cm) were fabricated by spreading 10% (w/v) PVDF solution containing 26.5 mg/cm^2^ of activated carbon (Norit SX-Plus, Holland) and 8.8 mg/cm^2^ of carbon black (Vulcan XC-72, Cabot Corporation, USA) directly onto a stainless steel mesh (1 mm × 1 mm, type 304, Eggs, TAIHO, Co, Japan). The air-cathode MFC was inoculated with activated sludge obtained from the Sapporo Sosei wastewater treatment plant (Sapporo, Japan) and continuously fed with the primary clarifier effluent (hereafter termed as “domestic wastewater”) at hydraulic retention time (HRT) of 1.5 h for more than one year to achieve the stable performance^16^. After confirming that the power generation became stable, a newly designed low voltage booster (LVB) with different numbers of AC/DC voltage multiplier circuits was connected with the MFC to evaluate its MFC energy harvesting performance.

### Low voltage booster multiplier (LVBM) electronic circuit

**Figure 1:**
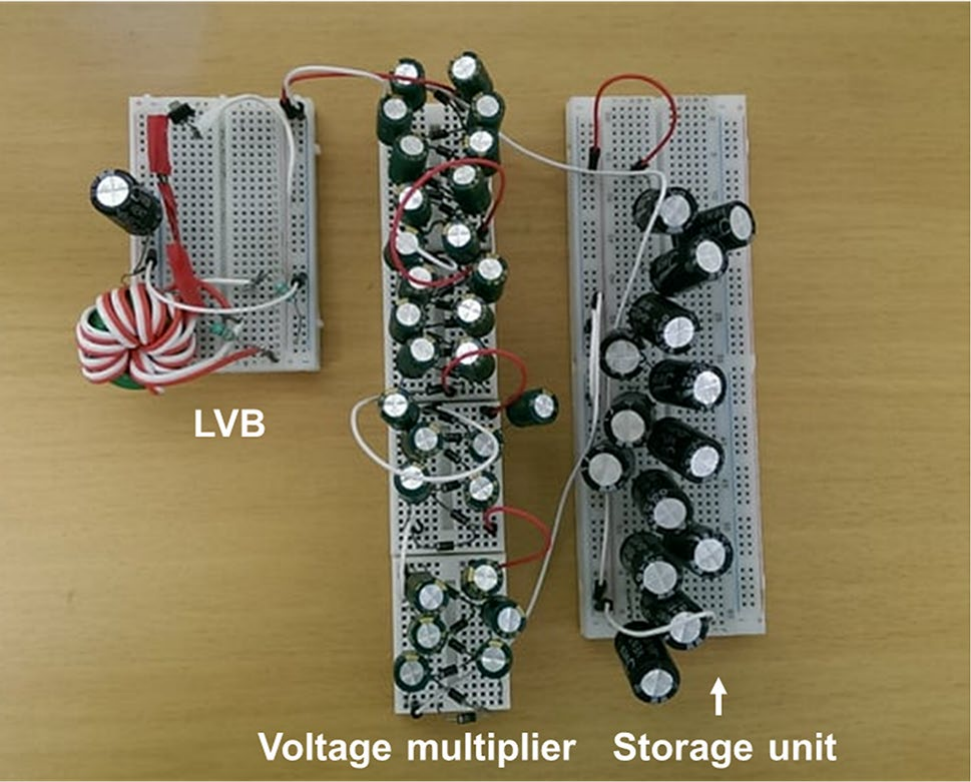
Photo of the newly designed low voltage booster multiplier (LVBM) system.

**Figure 2:**
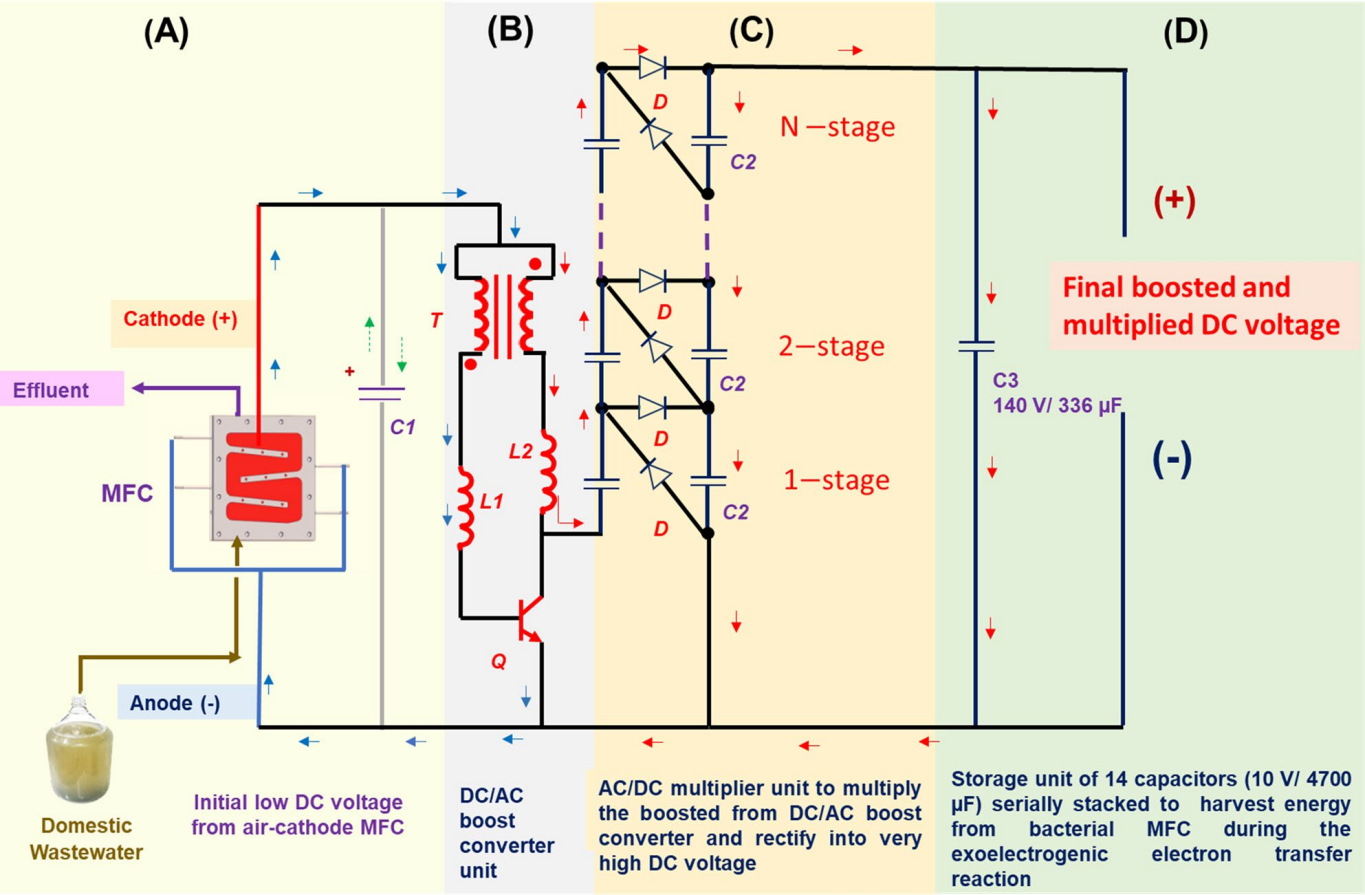
Diagram of the newly designed low voltage booster multiplier (LVBM) system connected to a single air–cathode microbial fuel cell (MFC) fed with domestic wastewater as substrate. (**A**) Air–cathode MFC fed with domestic wastewater, the brown and violet arrows indicate the influent and effluent, respectively. (**C1**) is a polarized capacitor (10 V/4700 µF). (**B**) Self-oscillating low voltage booster electronic circuit connected to the air–cathode MFC, **T** is a toroid ferrite coil inductor with double windings of 20 Turns, (**L1** and **L2**) are axial inductors (680 µH), **Q** is a superfast switch NPN transistor (STN851). (**C**) Half wave Cockcroft-Walton voltage multiplier circuit, **C2** is a polarized capacitor (6 V/1500 µF), **D** is a diode rectifier 1N4001. **(D)** Storage unit circuit, **C3** is an equivalent capacitor of 10 polarized capacitors (10 V/4700 µF) serially stacked. The blue arrows represent the current flow when **Q** is ON, and the red arrows represent the current flow when **Q** is OFF. The green dotted arrows represent the charge and discharge of capacitor **C1**.

In this study, a LVBM system was newly developed to boost and multiply the low voltage from a single air–cathode MFC. The LVBM is composed of a self-oscillating transistor-based LVB^16^, a multistage AC/DC voltage multiplier circuit, and a storage unit (Fig. 1). First, the low output DC voltage of MFC was boosted to an AC voltage via a direct current to alternating current (DC/AC) voltage boost converter composed of a low voltage fast-switching NPN power transistor (STN851)^19^ and inductors in which energy is charged and discharged into the circuit repeatedly (Fig. 2). This repeated charge and discharge cycles of the inductors enable the transistor STN851 to turn ON and OFF the circuit at a high frequency, thus inducing a high AC voltage spike (> 2 V). In this study, the transistor STN851 was selected because of its outstanding fast-switching speed and its very low saturation voltage between collector and emitter^19^ as compared to other bipolar junction transistors (BJTs) or metal–oxide–semiconductor field-effect transistors (MOSFETs) commonly used as a switch^16^.

Second, the boosted AC voltage is multiplied and converted to a DC voltage output via a multistage half-wave Cockcroft-Walton voltage multiplier circuit, which is a multistage single-phase cascade circuit of diodes and capacitors acting as a charge pump circuit at each stage (Fig. 2C)^17,18^. The AC voltage is rectified to DC voltage by the diode, and the current was stored in the capacitors. Therefore, the following voltage conversion flow is given;

$$\begin{aligned} & {\text{Low}}\;{\text{DC}}\;{\text{output}}\;{\text{voltage}}\;{\text{of}}\;{\text{MFC}} \to {\text{AC}}\;{\text{boosted}}\;{\text{voltage}} \to {\text{Multiply}}\;{\text{AC}}\;{\text{boosted}}\;{\text{voltage}} \times {\mathbf{n}} \\ & \to {\text{the}}\;{\text{boosted}}\;{\text{and}}\;{\text{multiplied}}\;{\text{AC}}\;{\text{voltage}}\;{\text{is}}\;{\text{rectified}}\;{\text{to}}\;{\text{DC}}\;{\text{voltage}} \to {\text{the}}\;{\text{electrical}}\;{\text{energy}}\;{\text{was}} \\ & {\text{stored}}\;{\text{in}}\;{\text{the}}\;{\text{capacitors}}.{\text{ }} \\ \end{aligned}$$

where **n** is the number of multiplying stages. The LVBM system can work even at low input current (ca. 1 mA) and voltage (ca. 0.4 V) without any external energy input.

Measurements of the current I (A) and voltage (V) of the MFC were performed every 30 min using a data logger (Agilent 34970A) and recorded in a personal computer. The electrode potentials were measured against an Ag/AgCl reference electrode (*E*_*0*_ = 195 mV vs. SHE, RE-1B, ALS Co, Japan).

### LVBM energy efficiency

The energy efficiency (*η*) of the LVBM system is defined as the ratio of the energy stored in the supercapacitor (*E*_*Cap*_) and the energy consumed (*E*_*MFC*_) when the MFC was used to charge the supercapacitor (Eq. (1)) as previously described^2,14,15^.

1 $$\eta = { }\frac{{E_{Cap} }}{{E_{MFC} }}$$

where *E*_*Cap*_ is the energy stored when the supercapacitor of capacitance C (Farad) was charged from 0 V to V Volt and is calculated as follows:

2 $$E_{{Cap}} = {\raise0.7ex\hbox{$1$} \!\mathord{\left/ {\vphantom {1 2}}\right.\kern-\nulldelimiterspace} \!\lower0.7ex\hbox{$2$}}\;{\text{C}} \times {\text{V}}^{2}$$

*E*_*MFC*_ is calculated as the integral of the product of electrical current produced by the MFC (*I*_*MFC*_) and the MFC voltage (*V*_*MFC*_) over the supercapacitor charging time *T* as given by Eq. (3)^2,15^.

3 $$E_{MFC} = \mathop \int \limits_{0}^{{\text{T}}} I_{MFC} V_{MFC} {\text{dt}}$$

To evaluate the energy harvesting efficiency, the MFC-LVBM system was used to charge five supercapacitors (2.7 V/350 F) set in series. The serially stacked supercapacitors have an equivalent capacitance of 70 F and a total voltage of 13.5 V. The output voltage of the array of supercapacitors, the MFC current, and the output current (current after voltage boosting) were measured every 2 min with the data logger. The energy efficiency test was performed in duplicate.

## Results and discussion

**Figure 3:**
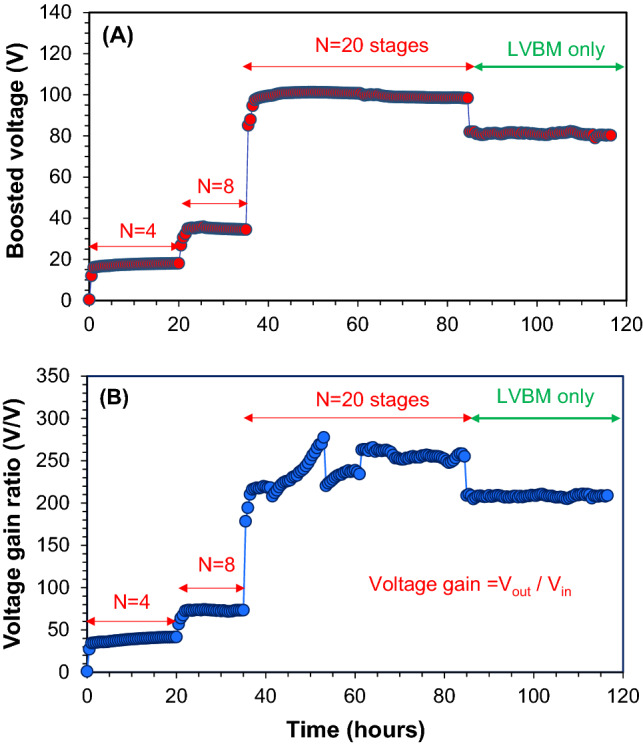
(**A**) Boosted voltage when the air–cathode MFC is connected to the LVBM with 4-stage, 8-stage, and 20-stage multiplier circuits, respectively. (**B**) DC–DC voltage gain when the air–cathode MFC is connected to the LVBM system. The green arrow indicates the voltage of the LVBM when the LVBM was disconnected from the MFC.

**Figure 4:**
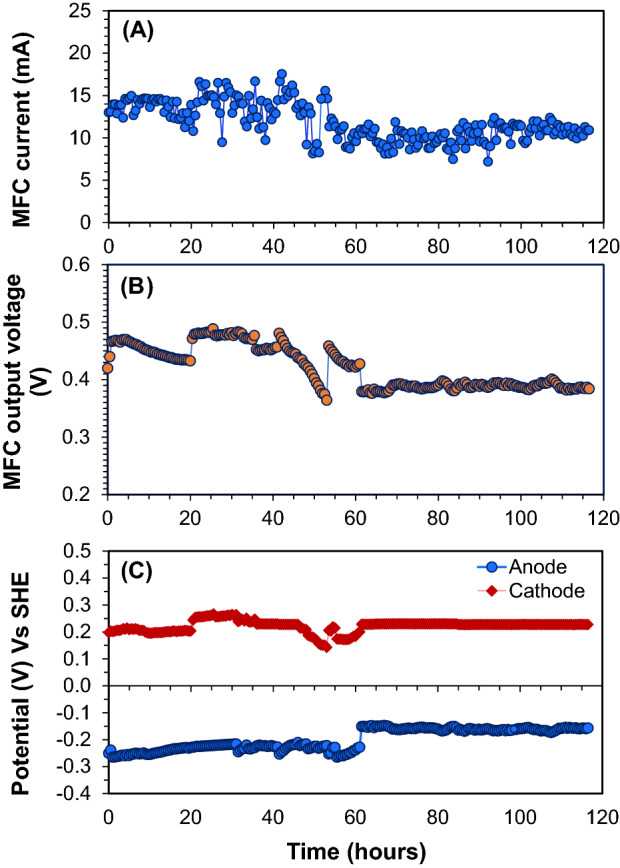
Time course of the produced current (**A**), the output voltage (**B**), and the electrode potentials (vs. SHE) (**C**) of the air–cathode MFC when the MFC was connected to the LVBM with 4-stage, 8-stage, and 20-stage multiplier circuits, respectively.

The air–cathode MFC was continuously operated at a COD loading rate of 6.6 ± 0.7 kg COD m^-3^ d^-1^ (1.5 h-HRT), and an average COD removal rate of 5.11 ± 0.94 kg COD m^-3^ d^-1^ was achieved (Figs. S1A, S1B). The power generation fluctuated due to unstable microbial activity and the fluctuation of influent COD concentrations, and the air–cathode MFC generated the average current density of 21.48 ± 16.97 mA m^-3^ and power density of 3.96 ± 3.01 W m^-3^, respectively (Figs. S2A, S2B). A newly designed LVBM was connected to a single air–cathode MFC, and its performance was evaluated. The average output voltage of the MFC during the whole experimental period was 0.45 ± 0.03 V. A 4-stage AC/DC voltage multiplier circuit was connected as an extension of the LVB electronic circuit (Fig. 2A) for the initial 20 h (Fig. 3A). The voltage immediately increased to 17 ± 1 V (Fig. 3A). Connecting an 8-stage AC/DC voltage multiplier circuit further increased the voltage to 35 ± 2 V. Furthermore, when the number of AC/DC voltage multiplier circuit was increased to 20 stages, the boosted voltage reached 99 ± 2 V (a maximum voltage was 101.24 V) and was kept stably at this level for 50 h (Fig. 3A), which was corresponding to about 243-hold DC to DC voltage gain (Fig. 3B). The performance of the air–cathode MFC, such as the produced current, the output voltage, and the electrode potentials (vs. SHE) was very stable during the entire experimental period (Fig. 4A,B,C).

After 85-h operation, the LVBM was disconnected from the MFC, but the stable LVBM output voltage of 81 ± 0.6 V was maintained for 40 h (Fig. 3B). This result demonstrated that since the LVBM system composed of a storage unit circuit (C3 in Fig. 2D) and serially stacked 10 polarized capacitors (10 V/4700 µF)), it could be used to store sufficient energy to power the electronic devices when the MFC voltage source is temporarily unavailable or widely fluctuated due to unstable microbial activity and influent organic loads.

Power management systems (PMSs) for MFCs are attracting considerable attention recently. It is essential to increase the output voltage and store the power when the extracted energy is used to drive any electronic devices in practice. Various self-starting PMSs have been designed, proposed, and tested for their performance. Although some improvements have been reported, the resulting boosted voltages were able to power only low-power electronics devices such as small wireless sensors or LEDs^2,6,7,10,13–15,20–22^ (Table 1). The highest boosted voltage reported to date was 12 V, which was harvested from a benthic MFC (V_MFC_ = 0.35 to 0.5 V) using two in-line flyback converters^13^.

The newly designed LVBM in this study is based on a unique two-step approach, by which the low DC voltage of MFC is boosted to AC voltage first, the AC boosted voltage is multiplied and converted to high DC voltage, and then the multiplied high DC voltage is stored. To our best knowledge, the boosted voltage (99 ± 2 V) in this study is the highest voltage that has ever been reported for a single air–cathode MFC treating low strength domestic wastewater (ca. 400 mg-COD L^−1^) so far (Table 1). For example, Kim et al.^6^ applied a commercially available LTC3108 voltage booster to a single air–cathode MFC fed with diluted swine wastewater (∼2 g COD L^−1^) as substrate and obtained a boosted voltage of 3.3 V^6^. In addition, Park and co-workers^14^ used a commercially available flyback voltage booster to increase the low voltage of a single air–cathode MFC fueled with sodium acetate (2 g L^−1^) and obtained a boosted voltage of 5 V.

**Figure 5:**
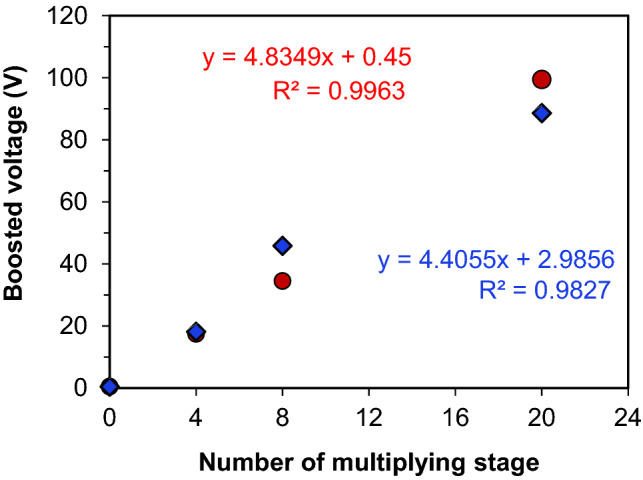
Correlation between the boosted voltages and the number of AC/DC voltage multiplier circuits applied. The number n = 0 indicates the output voltage of the single-chamber air–cathode MFC. The test was conducted in duplicate. The symbol (diamond) and (circle) represent the first and second boosted voltage test, respectively, showing rebroducible results.

Moreover, the newly designed LVBM system was found to be compatible with a low current range (7–17 mA) (Fig. 4A), which is usually produced by small lab scale MFCs. Thus, the LVBM system enabled us to boost the low MFC voltage to the required levels without voltage reversal because an only individual or parallel-connected MFCs is required to directly drive the DC/AC boost converter. In addition, since the boosted voltage is linearly correlated to the number of connected AC/DC voltage multiplier circuits (Fig. 5), we can design a LVBM which can supply the desired voltage by selecting the number of the multiplier circuit. However, the number of multiplying circuit was limited to 20 units for the safety reason in this study. Furthermore, the high voltage produced in this study is reproducible and even higher voltage > 100 V could be achieved if needed. The NPN transistor (STN851) used in the DC/AC boost converter can easily withstand high DC current up to 5A (at 25 °C operating temperature), which is the absolute maximum collector current rating (IC) (Table 2). This suggests that the NPN transistor-based LVBM system can be applied to a wide range of input current for large-scale practical MFC applications. For wastewater treatment application, several MFC units must be used to obtain a higher treatment capacity and power output. However, unlike conventional fuel cells, the overall power generation performance of stacked MFCs was not stable due to unstable or uneven microbial activity, which is generally limited by the worst performing unit(s). One of the solutions could be first to connect each MFC unit with a LVBM to increase or stabilize the individual power level and then connect all MFC-LVBM units in series or parallel. In this study, the MFC voltage was successfully boosted and amplified, but the current was still considerably low. In order to obtain enough power (*i.e*., current) to drive electronic devices, the MFC-LVBM must be connected in parallel. The feasibility study must be conducted in the future.

**Table 2:** Operating electrical characteristics of the LVBM system used in this study. I_CEmax_ is the theoretical maximum current supported by the LVBM system in case of large-scale application (i.e., an array of MFCs in parallel), V_CE max_ is the maximum voltage supported by the LVBM.

Symbol	Parameters	Value	Unit
I_CEmax_	Current collector-emitter max	5	A
I_Bmax_	Maximum base current	1	A
V_CE max_	Maximum voltage collector-emitter	60	V
I _EET_, _MFC_	Bacterial extracellular electron transfer (EET) range tested in this study to drive the LVBM system	0.8–17	mA
V_MFC_	MFC voltage operating range tested with the MFC	0.2–0.4	V
V_LVBM_	Voltage LVBM range obtained	17–102	V
V_LVBM_/V_MFC_	DC–DC voltage gain	Up to 240	V/V
Temperature	LVBM operating temperature range	15–25	°C
R_LVB_	Internal resistance of the LVB system only	91 ± 0	Ω
R_M_	Internal resistance of the voltage multiplier unit	N/A	Ω

N/A: Not available.

In this study, the LVBM was designed to harvest energy from low power generated by a single-chamber MFC without additional energy input to start. The energy harvesting efficiency of the LVBM was a key parameter to evaluate its performance and therefore evaluated using a 70-Farads 13.5 V supercapacitor and a single air–cathode MFC as a power supply (Fig. S4). In this experiment, the LVBM with a 4-stage AC/DC voltage multiplier circuit (LVBM-4) was used because the LVBM-4 could boost the voltage to 17 ± 1 V (Fig. 5), which is close to the nominal voltage of the supercapacitor.

**Figure 6:**
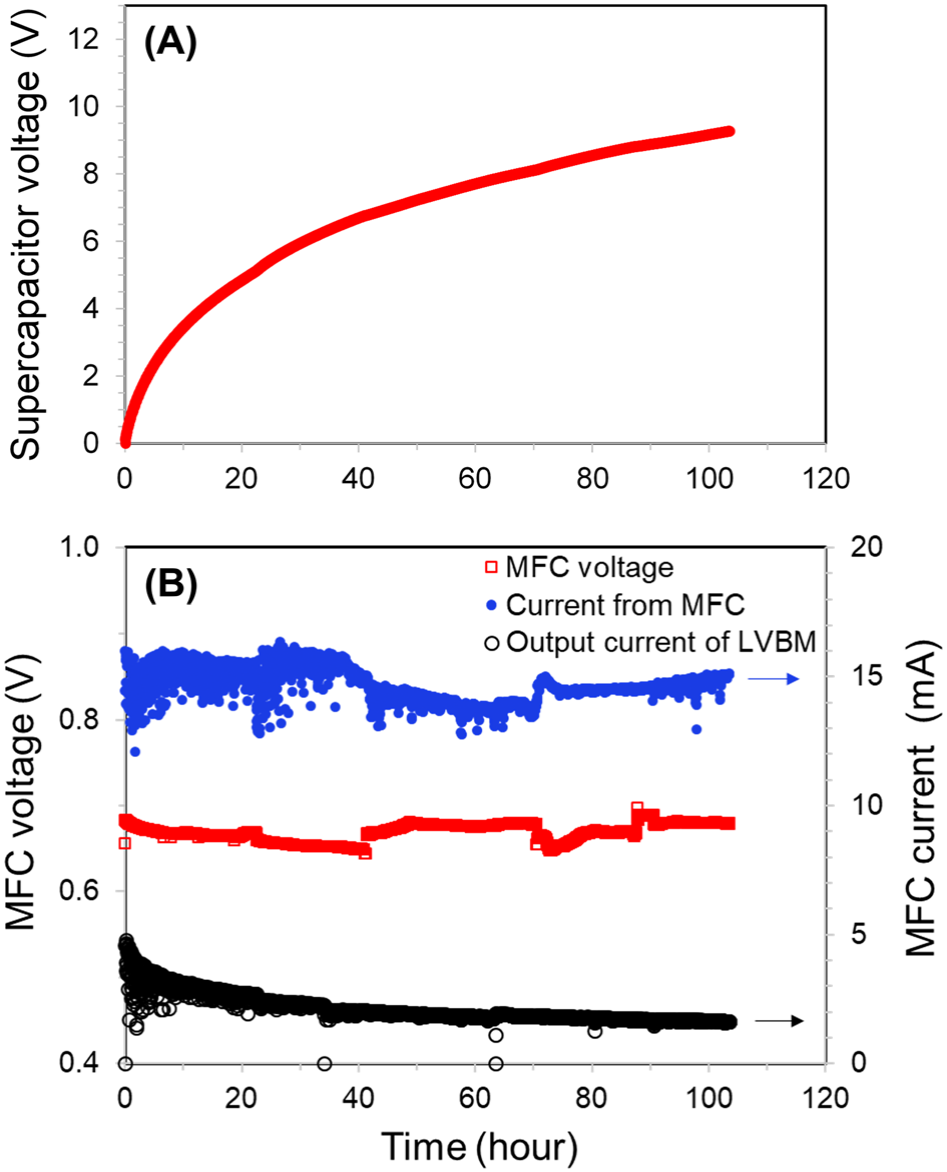
(**A**) Charging 70-F supercapacitor from 0 V to 9.3 V using a single-chamber air–cathode MFC as a power source with the LVBM. (**B**) Time course of current and voltage generated by the air–cathode MFC to power the LVBM system. The output current corresponds to the measured current after boosting the voltage with the LVBM system. MFC voltage is the output voltage when the air–cathode MFC is connected to the LVBM system to charge the supercapacitor.

The LVBM-4 charged the supercapacitor to 9.3 V in 103.5 h (Fig. 6A). The output voltage of the air–cathode MFC and the current flowing from the MFC to LVBM-4 remained unchanged (V_MFC_ = 0.66 ± 0.03 V with 14.7 ± 0.6 mA, respectively) while the supercapacitor was being charged. The output current of the LVBM-4 drastically decreased from14.7 ± 0.6 mA to 2.1 ± 0.5 mA (Fig. 6B). This indicates that the LVBM-4 charged the supercapacitor while receiving the input power of 9.82 ± 0.54 mW from the MFC. Accordingly, the energy harvesting efficiency of the LVBM-4 was calculated to 82.2% (Table S1). The energy harvesting efficiency was also determined while receiving relatively low input power of 0.25 ± 0.14 mW (V_MFC_ = 0.27 ± 0.08 V with 0.9 ± 0.21 mA) from the same air–cathode MFC. The LVBM-4 could harvest 83% of the MFC energy even when the input power is low (Table S1). This result suggests that the LVBM-4 can efficiently extract and store the relatively low energy from MFCs regardless of the input power.

The energy harvesting efficiency of LVBM-4 was higher than the previously reported values of capacitor-based DC-DC converters (5.33%)^2^, diode-based boost-converters (22%)^12^, and flyback converters (26–77%)^13,14^ (Table 1). This demonstrates that the LVBM has a highly efficient capacitor-charging performance. However, since the LVBM-4 extracted excessive current, the output current of the LVBM-4 was significantly reduced to 2.1 ± 0.5 mA (Fig. 6B).

Normalized energy recovery (NER_COD_) was proposed to evaluate the treatment performance in terms of COD removal and energy recovery^23,24^ (Table S2). The highest NER_COD_ in this study was 0.148 ± 0.007 kWh /kg COD, which is in agreement with the previously reported NER_COD_ values for MFCs fed with domestic wastewater as fuel^24,25^. The theoretical energy content in domestic wastewater was reported to be 3.86 kWh/kg COD in domestic wastewater^25^. Accordingly, the percentage of electrical energy recovered from real domestic wastewater is 3.83% (0.148/3.86 × 100 (%)) in this study. Furthermore, considering that the the energy harvesting efficiency of the LVBM-4 was 83%, the MFC-LVBM system retrieved 3.2% of the theoretically available energy in wastewater in this study.

The energy harvesting efficiencies could not be determined using the LVBM-8 and LVBM-10 (8 and 10 multiplying stages) because when the boosted voltage increased, the output current became extremely low (under the detection limit of the data logger). This suggests that increasing the output voltage with the LVBM is a trade-off between current reduction and overall power (energy) loss. The number of applied multiplying stages is highly dependent on the input power^17^ and thus must be optimized for individual MFCs to minimize the energy loss during the energy harvesting operation. The LVBM equipped with a number of multiplying stages could be applied for pilot-scale MFCs and MECs such as a biocathode MEC (1.5 m^3^) treating municipal wastewater that continuously generated a stable power output of 406 ± 30 mW m^−3^^26^. Possible applications of the LVBM system must be explored in the future.

In summary, a low voltage booster multiplier (LVBM) was newly designed for a single air–cathode MFC, and its performance was tested. The LVBM system was able to increase the low MFC output voltage (0.45 ± 0.03 V) to 99 ± 2 V and hold at this voltage level for 50 h without voltage reversal, which was more than 243-fold DC to DC voltage gain. The developed LVBM system was compatible with the low voltage and capable of handling widely fluctuated low current (0.8––17 mA) generated from low strength domestic wastewater. In addition, the LVBM output voltage was kept at 81 ± 0.6 V for 40 h even when disconnected from the MFC, indicating that the LVBM could be used as energy storage. The energy harvesting efficiency of the LVBM was > 82%. Since the LVBM is relatively cheap (less than $ 10) and easily assembled, the newly developed LVBM system is implementable as a self-starting low voltage booster or efficient energy harvester for MFCs.

## Supplementary information

Supplementary Information
