# Supplementary material for: High voltage generation from wastewater by microbial fuel cells equipped with a newly designed low voltage booster multiplier (LVBM)
Source: Sci Rep. 2020 Nov 4;10:18985. doi: 10.1038/s41598-020-75916-7 (PMC7642417; doi:10.1038/s41598-020-75916-7)
Supplement: Supplementary file 1 — Supplementary Information [file 41598_2020_75916_MOESM1_ESM.docx]

Supporting Information

**High voltage generation from wastewater by microbial fuel cells equipped with a newly designed low voltage booster multiplier (LVBM)**

**By**

**N’Dah Joel KOFFI and Satoshi OKABE***

*Division of Environmental Engineering, Faculty of Engineering, Hokkaido University,*

*North-13, West-8, Kita-ku, Sapporo, Hokkaido 060-8628, Japan*

* Corresponding Author

Satoshi Okabe

E-mail: sokabe@eng.hokudai.ac.jp

1. **COD removal performance of the air-cathode MFC**


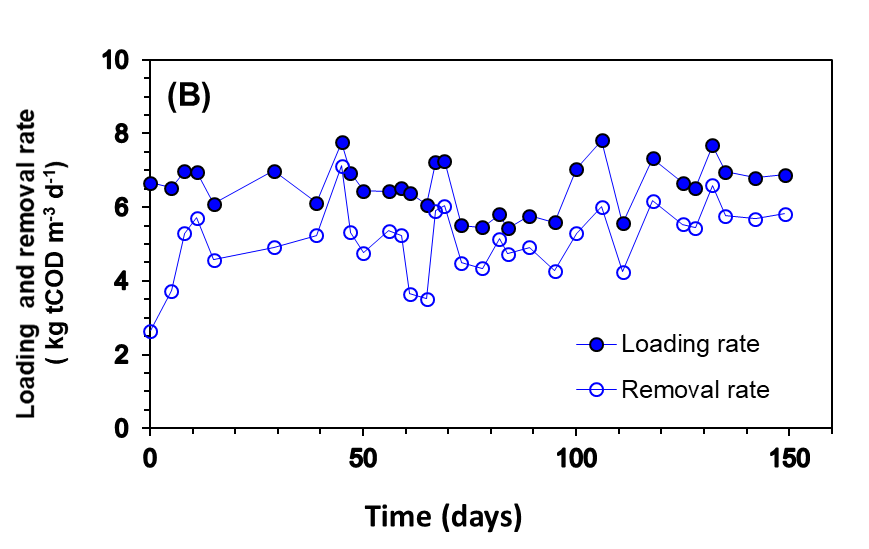

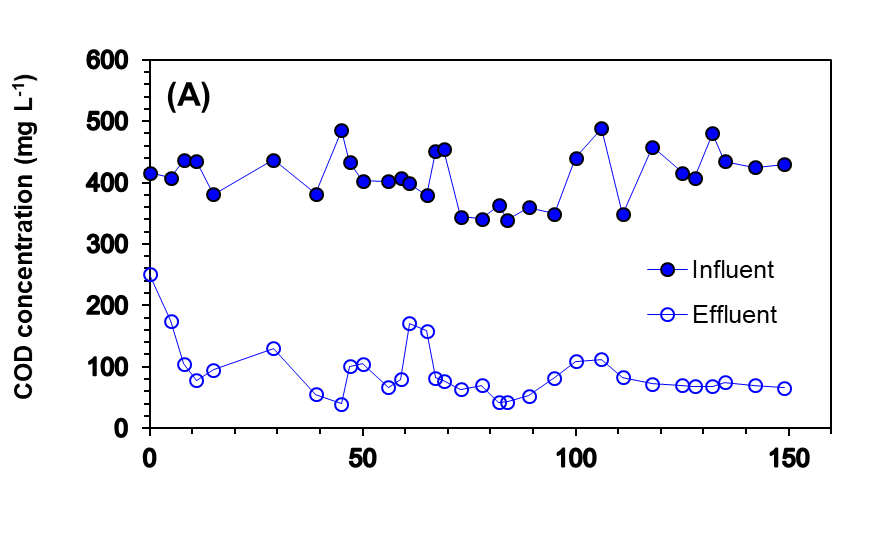


**Figure S1.** Time course of COD influent and effluent (**A**) and COD loading and removal rate (**B**) of the air-cathode MFC at 1.5 h HRT.

1. **Power generation performance of the air-cathode MFC**

**(A)**

**Figure S2.** Time courses of current density **(A)** and power density **(B)** generated by the air-cathode MFC.

1. **Polarization curve and supercapacitor charging experiment**


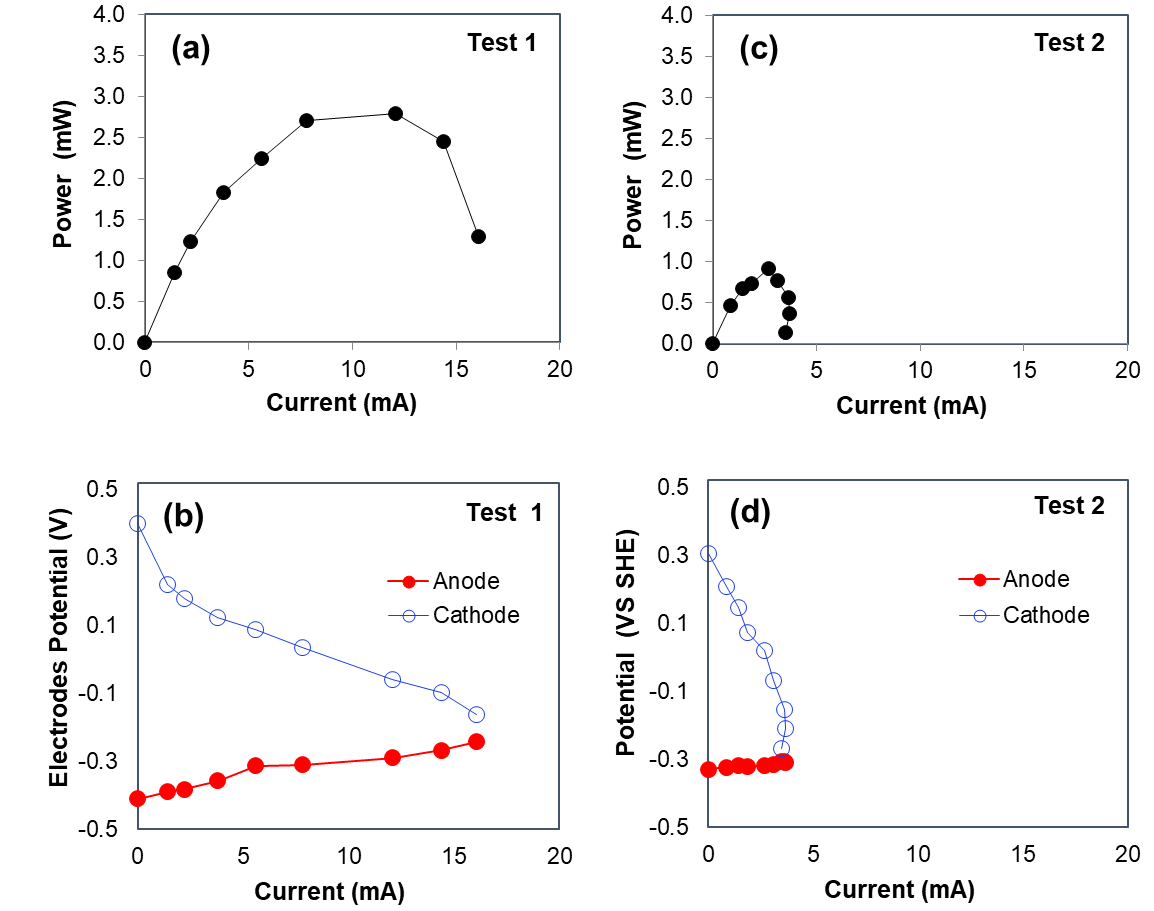


**(A)**

**(C)**

**(B)**

**(D)**

**Figure S3.**  Polarization tests were performed to evaluate the electrical performance of MFC. Power of the MFC and electrodes potential vs. SHE of the MFC during the first 70-Farad supercapacitor charging experiment (**Test #1**) **(A)** and **(B)**. Power of the MFC and electrodes potential vs. SHE of the MFC during the second 70-Farad supercapacitor charging experiment (**Test #2**) **(C)** and **(D)**.

**
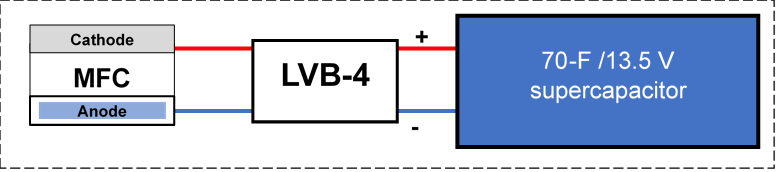
**

**Figure S4.** Experimental setup of a supercapacitor charging experiment.


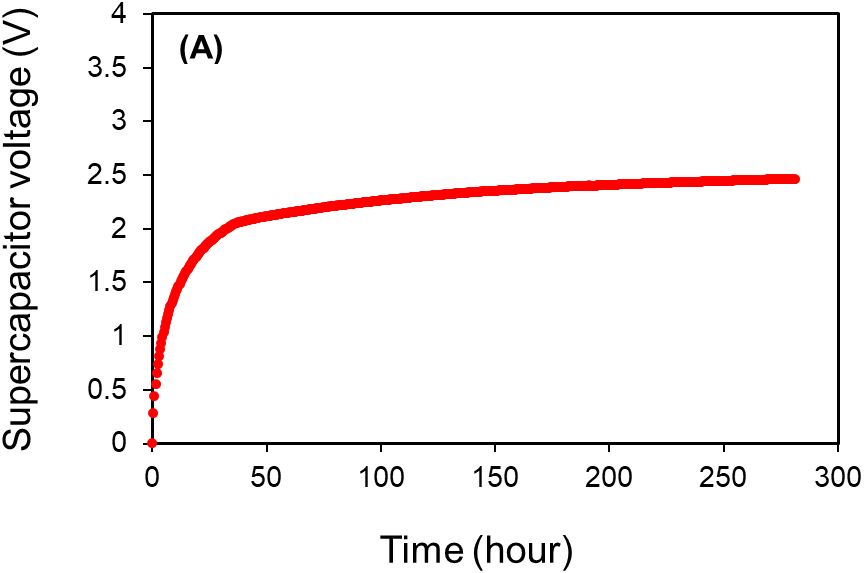

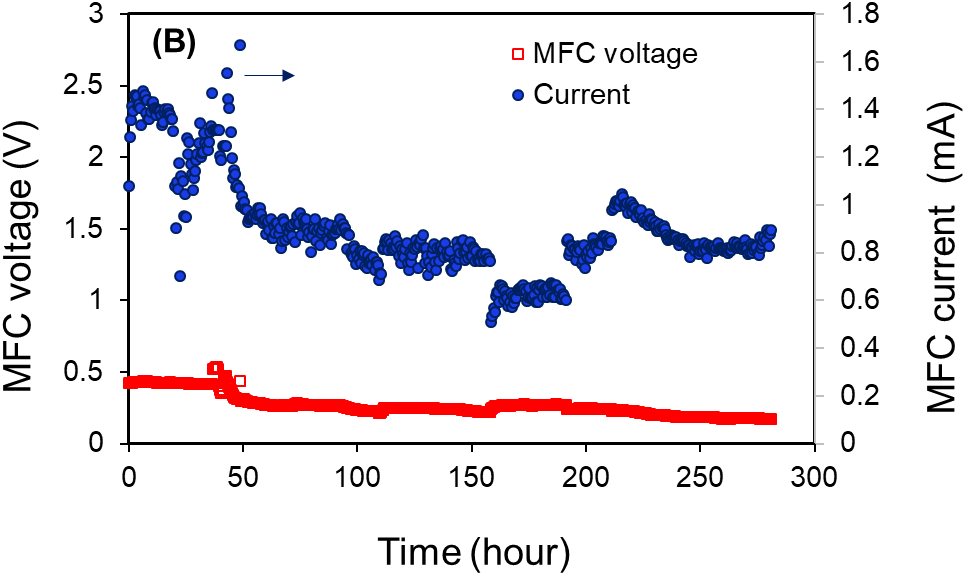


**Figure S5. (A)** Second charging 70-Farad supercapacitor test from 0 V to 2.47 V (**Test#2**) when using the single-chamber air-cathode MFC as a power source with the LVBM. **(B)** Time course of current and voltage generated by the air-cathode MFC to power the LVBM system. This experiment was performed when the MFC generated a relatively low current (0.90 ±0.21mA).

**Table S1** Summary of the energy efficiency tests of the LVBM system.

| Test Number | Power MFC (mW) | I (mA) | Input Energy (J) | *E_cap_* (J) | LVBM Energy Efficiency (%) | Charging time of supercapacitor (h) |
| --- | --- | --- | --- | --- | --- | --- |
| #1 | 9.82 ± 0.54 | 14.7 ± 0.64 | 3659 | 3008 | 82.2 | 103.5 |
| #2 | 0.25 ± 0.14 | 0.90 ±0.21 | 257 | 213 | 83.0 | 281 |

Test #1 and #2 were conducted to determine the energy harvesting efficiency of the LVBM system to charge a supercapacitor (70 F/ 13.5 V) from 0 V to 9.3 V and 0 V to 2.47 V, respectively. Values were average ± standard deviation. *E_cap_* corresponds to the energy harvested by the MFC and stored in the supercapacitor.

1. **Normalized Energy Recovery (NER) based on the wastewater treatment performances**

Normalized Energy Recovery (NER) was proposed to evaluate both the treatment performance in terms of COD removal and energy recovery ^1,2^. Contrary to the power density, the NER parameter eliminates the reactor dimensions factor and includes the wastewater flow rate and organic removal efficiency, which is relevant compared to two different MFC reactor sizes and configurations dedicated to wastewater treatment and energy recovery ^1^.

NER is expressed in two units, namely **NER_V_** or **NER_COD_** when the energy produced is relatively normalized based on the treated volume of wastewater or COD. The theoretical energy content in domestic wastewater is **3.86 kWh/ kg COD** oxidized to CO_2_ in domestic wastewater ^3^.

$\mathrm{NER}_{V}$ $=$ $\frac{Power ( kW)}{flow rate of wastewater \left( \frac{m^{3}}{h} \right)}$ (1)

$\mathrm{NER}_{\mathrm{COD}}$ $=$ $\frac{Power ( kW)}{flow rate of wastewater \left( \frac{m^{3}}{h} \right)\times\Delta COD(\frac{\mathrm{kg}}{m^{3}})}$ (2)

where $\Delta COD$is the difference between the influence and the effluent.

The NER of the air-cathode MFC during **Test #1** and **Test #2** are summarized in the table below:

Coulombic efficiency was calculated as CE = C_p_/C_th_ × 100%, where C_p_ is the total coulombs recovered by integrating the current over time, and C_th_ is the theoretical amount of coulombs available based on the COD removed in the MFC ^4^.

**Table S2.**  Normalized energy recovery of the air-cathode MFC during the supercapcitor Test #1 and Test#2. The MFC was fed at 1.5h-HRT (flow rate = 0.0002 m^3^/h).

| **Test #1** | | | | | |
| --- | --- | --- | --- | --- | --- |
| **Sampling time (h)** | **COD in (kg/m^3^)** | **COD out (kg/m^3^)** | **Coulombic Efficiency (%)** | **NER_V_**  **(kWh /m^3^)** | **NER _COD_ (kWh/kgCOD)** |
| 30 | 0.405 | 0.075 | 6.35 | 0.050 | 0.153 |
| 66 | 0.402 | 0.095 | 6.07 | 0.047 | 0.152 |
| 98 | 0.405 | 0.065 | 5.85 | 0.048 | 0.140 |
| **Average ± σ** | **0.4 ± 0.002** | **0.078 ± 0.015** | **6.1 ± 0.25** | **0.048 ± 0.002** | **0.148 ±0.007** |
| **Test #2** | | | | | |
| **Sampling time (h)** | **COD in (kg/m^3^)** | **COD out (kg/m^3^)** | **Coulombic Efficiency (%)** | **NER_V_**  **(kWh /m^3^)** | **NER _COD_ (kWh/kgCOD)** |
| 59 | 0.41 | 0.085 | 3.63 | 0.00128 | 0.03667 |
| 121 | 0.425 | 0.102 | 2.53 | 0.00050 | 0.01156 |
| 180 | 0.412 | 0.106 | 1.98 | 0.00050 | 0.01142 |
| 260 | 0.434 | 0.089 | 2.86 | 0.00050 | 0.01289 |
| **Average ± σ** | **0.42 ± 0.011** | **0.1 ± 0.01** | **2.75 ± 0.7** | **0.001 ± 0.0004** | **0.018 ±0.0124** |

The highest **NER_COD_** in this study (*i.e*., 0.148 ± 0.007 kWh /kg COD) in accordance with the typical value of **NER_COD_** reported with a typical MFC fed with domestic wastewater ^2^. The electrical energy recovery with air-cathode MFC treating real domestic wastewater is 3.84 % of the theoretical energy. Therefore, the MFC-LVBM system recovers 3.2 % of the theoretical available (LVBM-4 efficiency = 83%).

**References**

1. Xiao, L., Ge, Z., Kelly, P., Zhang, F. & He, Z. Evaluation of normalized energy recovery (NER) in microbial fuel cells affected by reactor dimensions and substrates. *Bioresource Technology* **157**, 77–83 (2014).

2. Ge, Z., Li, J., Xiao, L., Tong, Y. & He, Z. Recovery of Electrical Energy in Microbial Fuel Cells: Brief Review. *Environ. Sci. Technol. Lett.* **1**, 137–141 (2014).

3. McCarty, P. L., Bae, J. & Kim, J. Domestic Wastewater Treatment as a Net Energy Producer–Can This be Achieved? *Environ. Sci. Technol.* **45**, 7100–7106 (2011).

4. Logan, B. E. *Microbial fuel cells*. (Wiley-Interscience, 2008).
